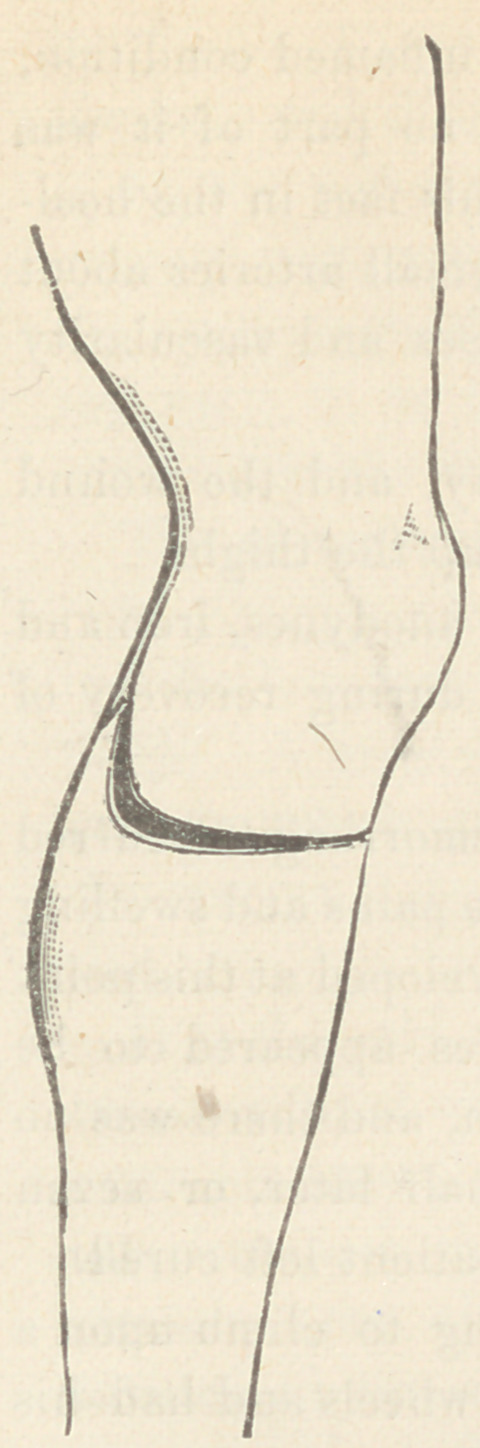# Surgical Clinic of Prof. Andrews

**Published:** 1881-10

**Authors:** 


### Clinical Reports.

### ARTICLE IX.

### SURGICAL CLINIC OF PROF. ANDREWS. AMPUTATION OF THE KNEE JOINT.

Amputation at the knee joint is an operation which has been unaccountably neglected in America and, in fact, the world over. Dr. Brinton, in 1868, published (*Am. Jour. Med. Sciences*) statistics of only four hundred cases of this amputation as a result of inquiries into the operations of the whole world. Ten years later I reported the search among the published statistics of both continents showing five hundred and fifty published cases. Since that time, although the operation has made progress in public favor, it has remained somewhat rare.

The following cases recently occurred in Mercy Hospital:

11,134. Female, aged 48. Entered hospital with a severe form of tertiary syphilis. The right leg was a mass of ulcers. The left one was similarly but less severely affected, being spotted with suppurating tertiary sores. Similar ulcers existed on other parts of the body to a limited extent.

The treatment consisted of iodide of potassium and local antiseptic dressings for twenty days, when it was found impossible to save the limb and it was amputated through the knee joint, after the method of Dr. Stephen Smith.

The following cut shows the line of external incision in the bilateral flap amputation recommended by him in his *Operative Surgery*:

Beginning about one inch below the tubercle of the tibia and, cutting to the bone the knife is carried downward and somewhat forward around the curve of the limb when it is turned somewhat backward and the incision terminates in an upward direction just beneath the line of articulation in the popliteal space. The flaps are similar except that the inner one is made somewhat more redundant to allow for the greater prominence of the inner condyle.

These flaps are made to include everything down to the bones, and when they have been carefully separated and raised, the ligaments of the joint are divided and the leg separated, only a scalpel being required to complete the cutting.

The older plan of removing the articular cartilages, or of sawing off the condyles is to be condemned in emphatic terms. In practice it is found that the much dreaded inflammations of the remaining joint surface is not met with if antiseptic treatment is effectively carried out. The patella is also left *in situ*, and never transposed to the fossa below as formerly recommended.

This patient left the hospital twenty-five days after the operation with the stump almost entirely healed by first intention.

Case 11,339. Male, aged 40. This man suffered from long standing necrosis in the upper part of the tibia of his right leg. In 1864, while a soldier, after great fatigue and exposure from sleeping upon the ground, he began to suffer from an inflamed knee and great lameness. This continued about one year, when an abscess formed upon the external aspect of the limb just below the joint, and there had been a running sore ever since. The head of the tibia was much enlarged and there was such extensive necrosis in its upper third that I agreed with the patient in preferring an amputation to an excision of the joint.

The amputation was performed as in the last case, leaving the patella which showed no implication in the disease, and the joint surfaces with all the cartilages of the femur. The external condyle of the femur showed plainly by its inflamed condition, that there had been synovitis; nevertheless, no part of it was removed, and not much trouble arose from this fact in the healing. Much time was taken to ligate all the small arteries about the joint, because of the excessive anastomoses and vascularity of the parts from the previous inflammation.

The operation was performed under spray, and the wound treated with Listerian dressings carried high up the thigh.

A very speedy recovery was obtained under anodynes, iron and cinchonidia, with a temperature at maximum during recovery of $101\frac{1}{2}°$ Fahrenheit.

Five weeks after the operation a slight hæmorrhage occurred from the wound, and at the same period severe pains and swelling over the outer condyle; a small abscess had developed at this point and was opened down to the bone. No caries appeared to be present. This opening was kept widely open, and there was no recurrence of the pain. Two weeks and a half later, or seven and one-half from the time of operation, the patient left cured.

Case 11,256. Male, aged 30. Attempting to climb upon a moving freight train, this man fell under the wheels and had his left leg crushed off at the upper third.

An hour after the accident his pulse was 120. An amputation was decided upon at the knee joint, after some hesitation in regard to the vitality of the tissues destined to compose the inner and external flaps. The skin and superficial fascia were found loosened at one part as high as the lower third of the thigh from their attachment to the parts within. Ultimately abscesses formed as high as the upper third, which caused some trouble but no serious delay in the healing.

Three hours after the accident the knee-joint amputation, as above described, was performed by Dr. E. Wyllys Andrews. The flaps were scanty and the external one bruised and torn.

Forty-eight hours later a small triangular segment of this flap was found to have mortified, and the wound gaped open at this point leaving the condyles of the femur exposed.

The temperature was for several days 103° at evening, and pulse 130.

The abscesses in the parts above were thoroughly carbolized by means of injections through a soft rubber catheter beneath the flaps and through the counter openings.

At the present time—seven weeks after the operation—the exposed parts are covered with rapidly-growing granulations, and the contraction of the cicatrix bids fair to draw in the surrounding parts so as to furnish a more or less complete tegumentary covering to the stump.

The advantages of this operation are such as to commend it most strongly where other indications are suitable. First of all, it is far less dangerous than amputation in the lower third of the thigh. A greater length of limb is at the same time secured and one which gives the best support to an artificial limb. In this respect it is superior even to the stump after a leg amputation.

It is the only stump upon which the entire weight of the body can be carried without pain or ulceration. The scar left is surprisingly small, and is in the posterior aspect where it does not interfere with the use of any artificial appliance. There is no cicatrix upon the end of the stump at all. No bone being divided in the operation, which requires for its execution only a strong scalpel, the danger of osteo-myelitis is not to be counted among the risks attending it. The supposed dangers from leaving in the wound a large synovial surface are from *a priori* conclusions and are wholly mythical.

---

NOCTURNAL INCONTINENCE OF CHILDREN.—Prof. S. D. Gross, of Philadelphia, advises the following formula:

| | |
|---|---|
| Strychniæ | gr. j. |
| Pulv. cantharides | grs. ij. |
| Morph. sulph | grs. iss. |
| Ferri. pulv | scr. j. |

MIX. Make 40 or 50 pills or powders, *pro re nata*. Sig.—One three times a day to a child ten years old.

This prescription will speedily relieve the irritability of the bladder, especially if conjoined with such means as a cold shower bath daily, the avoidance of irritant food and late suppers, the patient lying on the side or belly, and taking care to drink nothing for the few hours preceding sleep, and to empty the bladder on going to bed.—*Mich. Med. News.*

## Figures and Tables

**Figure f1:**